# Molecular Mechanisms of Disease Progression in Primary Cutaneous Diffuse Large B-Cell Lymphoma, Leg Type during Ibrutinib Therapy

**DOI:** 10.3390/ijms19061758

**Published:** 2018-06-13

**Authors:** Lucy C. Fox, Costas K. Yannakou, Georgina Ryland, Stephen Lade, Michael Dickinson, Belinda A. Campbell, Henry Miles Prince

**Affiliations:** Department of Hematology, Peter MacCallum Cancer Centre, Melbourne, VIC 3000, Australia; lucy.fox@petermac.org (L.C.F.); costas.yannakou@petermac.org (C.K.Y.); georgina.ryland@petermac.org (G.R.); stephen.lade@petermac.org (S.L.); michael.dickinson@petermac.org (M.D.); belinda.campbell@petermac.org (B.A.C.)

**Keywords:** primary cutaneous diffuse large B-cell lymphoma, leg type, ibrutinib

## Abstract

Primary cutaneous diffuse large B-cell lymphoma, leg type (PCDLBCL-LT) is one of the well-recognized extranodal lymphomas commonly addicted to the B-cell receptor-MYD88 superpathway. We aimed to describe the genomic changes in a patient who progressed through treatment with ibrutinib, a Bruton’s tyrosine kinase (BTK) inhibitor. An 80-year-old woman presented with multiply relapsed PCDLBCL-LT after multiple lines of chemoimmunotherapy and radiotherapy. Pre-treatment testing of the localized cutaneous tumor lesion on a lymphoid amplicon panel demonstrated an *MYD88* p.L265P mutation. Ibrutinib therapy was subsequently commenced, resulting in complete resolution of the skin disease. Despite an ongoing skin response, the patient developed progressive nodal disease at two months. Genomic analysis of the cutaneous tumor sample at baseline was compared to that of the inguinal lymph node upon progression, and revealed the acquisition of multiple genomic changes. These included several aberrations expected to bypass BTK inhibition, including two *CARD11*-activating mutations, and a deleterious mutation in the nuclear factor kappa B (NF-κB) negative regulator, *NFKBIE*. In addition, an IgH-IRF8 translocation was detected (which brings the IRF8 transcription factor under control of the immunoglobulin heavy chain locus), representing a third plausible mechanism contributing to ibrutinib resistance. Several copy-number changes occurred in both samples, including an amplification of 18q, which encodes the anti-apoptotic protein BCL2. We describe the first case of novel genomic changes of PCDLBCL-LT that occurred while on ibrutinib, providing important mechanistic insights into both pathogenesis and drug resistance.

Primary cutaneous diffuse large B-cell lymphoma, leg type (PCDLBCL-LT) is recognized in the World Health Organization classification of hematological malignancies [1]. It predominately affects elderly women, usually involving the lower leg; however, it may arise elsewhere, and frequently disseminates to extracutaneous sites. Mutational and cytogenetic analyses suggest that the cell of origin subtype of PCDLBCL-LT tumors is more in keeping with the activated B-cell-like (ABC) type of diffuse large B-cell lymphomas, than with the germinal center B-cell-like (GCB) type [2].

Recent studies described rates of *MYD88* p.L265P mutations in patients with PCDLBCL-LT of up to 69% [3], where it was associated with inferior patient outcomes [4]. Activating *MYD88* mutations promote the survival of ABC DLBCL cells lines through the nuclear factor kappa B (NF-κB) pathway, which in turn can be blocked through inhibition of Bruton’s tyrosine kinase (BTK) (which links B cell receptor activity to NF-κB transcription). [5]. Ibrutinib, a BTK inhibitor, was demonstrated to be effective in patients with ABC DLBCL, particularly those with *CD79B* and *MYD88* co-mutations [5].

We report the case of an 80-year-old woman who presented with a localized subcutaneous nodule in her right axillary fold in May 2012. Histopathology following a core biopsy demonstrated sheets of large cells staining for pan-B cell markers CD5, BCL2, and MUM1, but not for CD10, which in the absence of nodal and extracutaneous disease was diagnostic of PCDLBCL-LT. She received six cycles of R-CHOP chemoimmunotherapy (rituximab, cyclophosphamide, doxorubicin, vincristine, and prednisolone), and attained a complete metabolic response (CMR) on restaging ^18^F-FDG-PET after 3 cycles. She remained in remission until October 2014, at which point she presented with a lesion on her upper left thigh. A biopsy confirmed relapsed PCDLBCL-LT (CD20, PAX5, MUM1, FOXP1, and BCL2 positive; and CD10 and BCL6 negative), and she was subsequently treated with radiotherapy only.

She remained in second remission until January 2016, at which point she developed further lesions on her left thigh. Biopsy confirmed relapsed PCDLBCL-LT, and an ^18^F-FDG-PET scan demonstrated the presence of three soft-tissue nodules in her left lower limb. Systemic therapy with rituximab, and reduced-dose gemcitabine and vinorelbine was administered, with subsequent radiotherapy resulting in a partial response. In July 2016, she developed a papule on her left thigh at the margin of the previous radiotherapy field. A third course of radiotherapy was administered, and the patient was commenced on lenalidomide. A further short remission ensued, and in both September and October 2016, new lesions developed outside the previous radiotherapy fields, for which she underwent a fourth course of radiotherapy. An ^18^F-FDG-PET scan in December 2016 demonstrated complete response in irradiated areas, but also a progression of the disease in her left thigh outside the radiation fields, and nodal progression within the pelvis. 

A biopsy of the new cutaneous nodule in her left thigh confirmed PCDLBCL-LT, and for the first time, DNA analysis was performed. Routine testing on a custom-designed lymphoid 26-gene amplicon panel demonstrated an *MYD88* p.L265P missense mutation. More extensive analysis was undertaken using a custom hybridization capture panel developed in-house, comprising 313 genes of interest in hematological malignancies, demonstrating a *CD79B* p.Y196H mutation.

The patient was commenced on ibrutinib (420 mg daily), with subsequent complete resolution of the skin changes. Two months later, she presented with lymphedema in her left leg, and imaging demonstrated a discordant response to ibrutinib, with progressive inguinal, iliac, and paraaortic nodal disease, and an ongoing complete cutaneous response.

A biopsy of an inguinal lymph node in February 2017 confirmed refractory PCDLBCL-LT, and DNA sequencing again demonstrated the previously detected *MYD88* and *CD79B* mutations. However, additional genetic abnormalities were detected in this post-ibrutinib sample, potentially representing mechanisms of ibrutinib resistance in the progressive nodal disease (see Table 1). Two *CARD11* mutations were detected, and were confirmed to be absent from the pre-ibrutinib cutaneous sample after manual review of the sequence reads. The emergence of *CARD11* mutations in the progressive nodal post-ibrutinib biopsy was in keeping with previous observations that ibrutinib was ineffective in ABC DLBCL patients with activating *CARD11* mutations [5]. In particular, the p.K215M mutation occurs within the CARD11 coiled-coil domain, the same domain in which the mutations possibly conferring ibrutinib resistance were previously described. Of note, *CARD11* mutations have been described only rarely in PCDLBCL-LT [6].

A further new abnormality detected in the post-ibrutinib sample was a p.G460A mutation in NFKB inhibitor epsilon (*NFKBIE*), a negative regulator of NF-κB, representing a further plausible mechanism of ibrutinib resistance. Normally, NFKBIE levels are upregulated following NF-κB activation, and its usual role is to retain NF-κB in the cytoplasm with subsequent inhibition of NF-κB-directed gene expression [7]. We postulate that the *NFKBIE* mutation observed in this patient was inactivating, resulting in a lack of cytoplasmic retention of NF-κB, with subsequent increased NF-κB-directed transactivation.

A further potential mechanism of drug resistance was the translocation of (14;16)(q32.33;q24.1) (IgH-IRF8), observed in the post-ibrutinib sample only. This translocation brings the IRF8 transcription factor under the control of the immunoglobulin heavy-chain locus, thereby causing its dysregulation. We examined IRF8 expression in both the pre-ibrutinib cutaneous sample and the post-ibrutinib nodal sample (Figure 1). While acknowledging the limitations of comparing antigen expression across different tissue types, we observed increased IRF8 staining in the post-ibrutinib sample, possibly reflecting IRF8 upregulation caused by the demonstrated translocation. Pre-ibrutinib IRF8 staining was mild–moderately positive in 70% of cells, whereas in the post-ibrutinib sample, IRF8 staining was strongly positive in over 95% of cells. The IgH-IRF8 gene fusion has been described once previously in a patient with CD5 positive GCB DLBCL [8].

Off-target reads from the hybridization capture panel were used to determine low-coverage whole-genome copy-number variation, visualized using a browser developed in-house. This analysis provided insight into disease pathogenesis, with several abnormalities being detected in both the pre- and post-ibrutinib samples (Figure 2). Amplification of a region on chromosome 18q, harboring the gene encoding anti-apoptotic protein BCL2, was observed. Another gene implicated in this amplification was *TNFRSF11A*, which encodes a receptor involved in NF-κB activation via both canonical and non-canonical pathways [9]. Additionally, both samples contained two distinct homozygous deletions of 9q, one of which involved the tumor suppressor *CDKN2A*, and the other which involved *PTRPD*, a protein tyrosine phosphatase which acts as a signaling molecule involved in cell-cycle regulation, and oncogenic transformation [10].

The patient underwent further radiotherapy with impressive regression of the left-leg lymphedema. She remained on ibrutinib daily for a further three months with no evidence of recurrence of cutaneous disease; however, she eventually succumbed to progressive disease in the pelvis and abdomen.

This case has several features consistent with previously understood mechanisms of disease: the pre-ibrutinib sample harbored mutations in both the B-cell receptor (BCR) subunit, *CD79B*, and also an *MYD88* p.L265P mutation conferring constitutive activation of NF-κB. These variants were also among a restricted set of recurrent mutations in PCDLBCL-LT, recently identified by Mareschal et al. [6], who described a unique mutational landscape underlying this disease. High rates of response to ibrutinib in ABC DLBCL patients with co-mutated *CD79B* and *MYD88* have been reported [5], providing the rationale for trialing ibrutinib therapy in this patient with PCDLBCL-LT. We note with interest, a case report of the successful treatment of multiply relapsed PCDLBCL-LT (mutant *MYD88*, and wildtype *CD79A/B*) with ibrutinib and chemotherapy (R-EPOCH—rituximab, etoposide, prednisolone, vincristine, cyclophosphamide and doxorubicin) with remission exceeding 20 months. This patient had neither *CARD11* nor *NFKBIE* mutations detected; however, had mutations in 18 additional genes, several of which (e.g., *STAT6* and *BARD1*) may have affected NF-κB activity [11].

Ibrutinib was prescribed until the pre-terminal phase of the illness, and we noted the ongoing cutaneous response to this agent. We believe that this patient exhibited spatial tumoral heterogeneity, with ibrutinib-resistance mutations identified in her nodal disease, but not in her cutaneous disease.

We describe the first case of evolving, novel genomic changes in PCDLBCL-LT that occurred while on ibrutinib, providing important mechanistic insights into both pathogenesis, and drug resistance. This highlights the existence of a dynamic mutational landscape, which may limit the efficacy of BTK inhibition in certain patients with this condition.

## Figures and Tables

**Figure 1:**
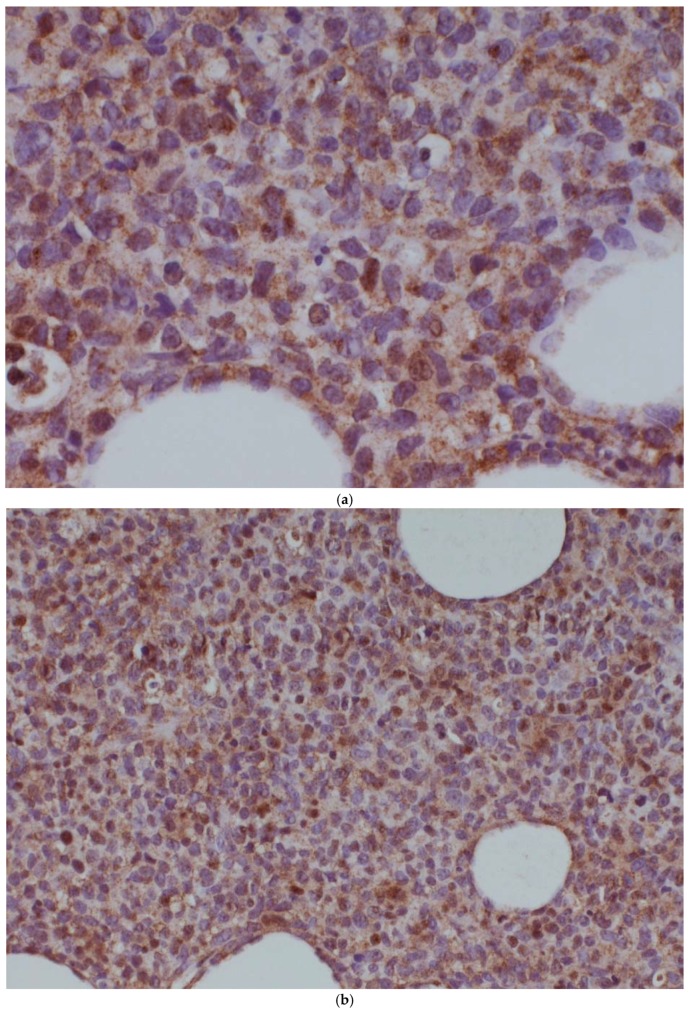

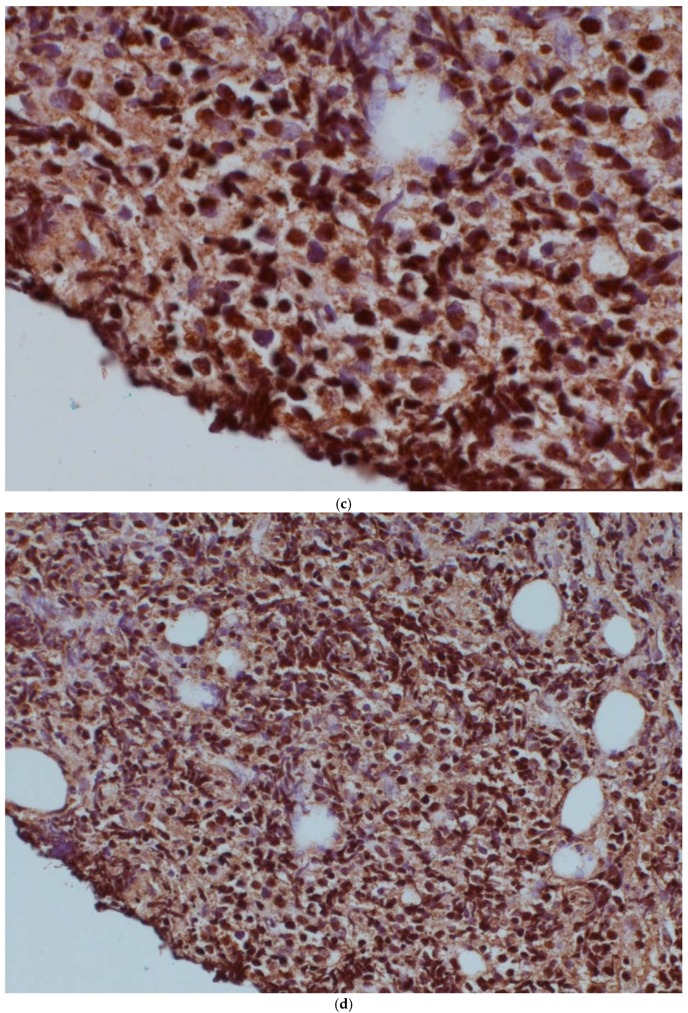
IRF8 expression in the pre-ibrutinib cutaneous lesion lacking t(14;16) (**a**,**b**), and post-ibrutinib nodal sample containing t(14;16) (**c**,**d**). Magnification times, (**a**,**c**) 100×, (**b**,**d**) 40×.

**Figure 2:**
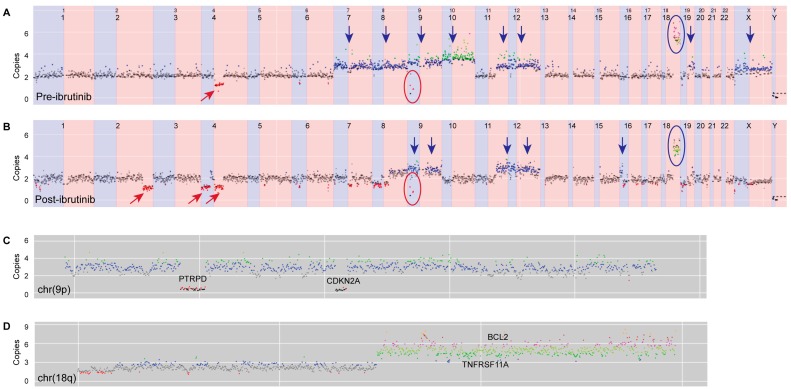
Copy-number changes in (**A**) pre- and (**B**) post-ibrutinib samples. Apparent changes are indicated as follows: red arrows—deletions, blue arrows—gains, red rings—homozygous deletions of 9p, and blue rings—amplification of 18q. Further details of post-ibrutinib copy-number changes in chromosomes 9p and 18q are shown in (**C**,**D**), respectively. Colored dots represent copy number (black = 0, red = 1, gray = 2, blue = 3, green = 4, olive = 5, pink = 6).

**Table 1:** Genomic alterations pre- and post-ibrutinib.

Genomic Alteration	Pre-Ibrutinib (Skin)	Post-Ibrutinib (Node)
Variants	*MYD88* c.794T > C;p.L265P	*MYD88* c.794T > C;p.L265P
	*CD79B* c.586T > C;p.Y196H	*CD79B* c.586T > C;p.Y196H
		***CARD11*** **c.367G > T;p.G123C**
		***CARD11*** **c.644A > T;p.K215M**
		***NFKBIE*** **c.1379G > C;p.G460A**
Copy-number changes	Gain **chr7, chr8,** chr9, **chr10**, 11q, chr12, **19q**, **chrX**	Gain 8q, chr9, 11q, chr12, 16p
	Amplification 18q (*BCL2/TNFRSF11A*)	Amplification 18q (*BCL2/TNFRSF11A*)
	Del 4q	**Del 2q**, **4p**, **4q**, **6p**, **7q**, **8p**, **8q**, **16p**, **16q**, **17p**, **17q**, **19p**,
	Homozygous deletion 9p containing *CDKN2A* Second homozygous deletion 9p containing *PTRPD*	Homozygous deletion 9p containing *CDKN2A* Second homozygous deletion 9p containing *PTRPD*
Translocation	Not detected	**t(14;16)(q32.33;q24.1) IgH-IRF8**

1. Bold font indicates a difference between both samples. 2. Mutations are described according to the following transcripts: *MYD88* NM_002468.4, *CD79B* NM_000626.2, *CARD11* NM_032415.4, and *NFKBIE* NM_004556.2.
